# Age‐Associated Impairment of Paneth Cells Driven by microRNA‐152 Promotes Intestinal Epithelial Vulnerability to Pathological Stress

**DOI:** 10.1111/acel.70542

**Published:** 2026-05-13

**Authors:** Bridgette Warner, Haonan Zhao, Hongxia Chen, Amy VanderStoep, Ana S. G. Cunningham, Dongyoon Yoo, Hee K. Chung, Christine Brantner, Jennifer Coleman, Joungil Choi, Rosemary Kozar, Myriam Gorospe, Jian‐Ying Wang, Lan Xiao

**Affiliations:** ^1^ Cell Biology Group, Department of Surgery University of Maryland School of Medicine Baltimore Maryland USA; ^2^ Veterans Affairs Maryland Healthcare System Baltimore Maryland USA; ^3^ Department of Oncology and Diagnostic Sciences University of Maryland School of Dentistry Baltimore Maryland USA; ^4^ Pathology Biorepository Shared Resource University of Maryland School of Medicine Baltimore Maryland USA; ^5^ Shock Trauma Center University of Maryland School of Medicine Baltimore Maryland USA; ^6^ Laboratory of Genetics and Genomics National Institute on Aging‐IRP, NIH Baltimore Maryland USA; ^7^ Department of Pathology University of Maryland School of Medicine Baltimore Maryland USA

**Keywords:** aging, gut mucosal defense, microRNAs, mitochondrial function, Paneth cells, PHB1

## Abstract

Advanced age is a well‐known risk factor for severe complications in surgical patients with critical illnesses, partly due to declined intestinal mucosal defense, but the underlying mechanism remains largely unknown. Our study provided direct evidence from older human and mouse small intestines of the adverse impact of aging on Paneth cell functionality by impaired mitochondrial metabolism. Mechanistic investigation revealed a specific elevation of Paneth cell enriched microRNA‐152 (miR‐152) in aging small intestinal epithelium. Increased miR‐152 impaired Paneth cells of aging small intestine by inhibiting mitochondrial protein Prohibitin1 (PHB1) expression and disrupting mitochondrial respiration. Moreover, the levels of *circHIPK3*, an intestinal circular RNA that counters the function of miR‐152, also declined in older intestines, further enabling miR‐152‐mediated deterioration in Paneth cell function. Conversely, antagonizing miR‐152 improved mitochondrial metabolism by restoring PHB1 levels and ameliorated the functional decline of old Paneth cells. Our findings indicate that dysregulated miR‐152 expression and activity play a central role in the loss of Paneth cell homeostasis in the aging small intestine, offering translational insights for protecting intestinal mucosal integrity in older patients.

## Graphical abstract

Aging associated miR‐152 dysregulation plays a central role in the loss of Paneth cell homeostasis by inhibiting mitochondrial protein Prohibitin1 (PHB1) expression, which contributes to increased small intestinal vulnerability to pathological stresses.

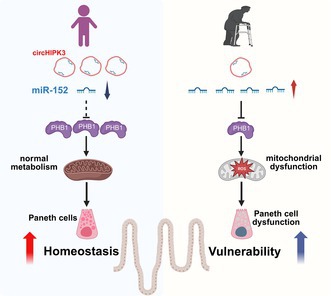

**Abbreviations**
circRNA: circular RNA
IEC: intestinal epithelial cell
miR: microRNA
ncRNA: noncoding RNA
Q: quantitative
RBP: RNA‐binding protein
RT: reverse transcription

## 1 Introduction

The process of aging is characterized by a progressive loss of physiological integrity of all organs, including the gut (Man et al. 2014; Parrish 2017; Funk et al. 2020; An et al. 2018). The systemic effects of aging gut are reported to be involved in an array of chronic disorders, such as diabetes, Parkinson's disease, and Alzheimer's disease (Mossad and Blank 2021; Sorini et al. 2019; Nicoletti 2015; Li et al. 2016). In patients with critical illnesses, the effect of advancing age is not well studied but is clinically recognized as a risk factor for infection, sepsis, and multiple organ dysfunction following trauma, burn, and major surgical intervention (Assimakopoulos et al. 2018; Brakenridge et al. 2018; Sakr et al. 2018; Starr and Saito 2014; Faries et al. 1998). Besides the altered immune function with aging—inflammaging, declined gut homeostasis and impaired intestinal mucosal defense are believed to facilitate infection and systemic inflammatory response under stressful critical conditions. However, our current understanding of the mechanisms underlying the decreased intestinal mucosal defense and increased gut vulnerability of advanced age remains poor and often inconsistent.

Gut barrier acts as a major frontline defense system to protect our internal environment by preventing the penetration of harmful entities, such as pathogens, luminal antigens, and proinflammatory factors (Hu and Jasper 2017; Camilleri 2019; Yang et al. 2014). A functional gut barrier requires concerted coordination from various intestinal components, including a physical barrier formed by the mucous layer, epithelium, and endothelium; an immunological barrier of immune cells, secretory IgA, anti‐microbial peptides and proteins (AMPs); and intestinal microbiota. In particular, the single layer of intestinal epithelial cells (IECs) that cover the entire gut luminal surface plays the most critical role by forming a mechanically tight barrier and an immunological defense via several specialized cell types such as Paneth cells.

Paneth cells are secretory IECs that reside exclusively in the small intestinal crypt base and play unique roles in maintaining intestinal epithelial integrity and innate immune defense. On the one hand, Paneth cells provide multiple secreted and surface‐bound niche signals such as Delta‐like ligand (Dll) and Epidermal growth factor (Egf) to adjacent intestinal stem cells, which are crucial to a constant process of epithelial renewal for homeostasis and mucosal repair under various pathophysiological conditions (Xiao et al. 2023; Nalapareddy et al. 2017; Quintero and Samuelson 2025). On the other hand, Paneth cells are the major source of AMPs such as Lysozyme and Defensin alpha (Defa), which are essential to clear luminal pathogens and regulate the composition of intestinal microbiota (Mukherjee and Hooper 2015; Salzman et al. 2010). Paneth cell activity is dynamically adjusted upon changes in the intestinal environment, immune cell‐derived cytokines, and IEC intrinsic factors. Recent studies show that there is a fundamental dependency of Paneth cell function on cellular metabolism and energy supply from mitochondria (Ho and Theiss 2022; Ray 2020). However, there has been limited research to directly address the challenges that old age places on the small intestinal Paneth cells.

In mammalian cells, posttranscriptional regulation is a crucial step in the control of gene expression (Xiao and Wang 2014; Choquet et al. 2025; Ilik et al. 2025). Extensive research has revealed a diverse group of functional RNA molecules without protein coding potential that play important roles in posttranscriptional gene control (Xiao et al. 2021, 2018; Li et al. 2020). The size of these non‐protein coding RNAs (ncRNAs) ranges from less than 20 nucleotides microRNAs (miRs) to several thousand long ncRNAs (lncRNAs). Among them, microRNAs are the most well‐studied ncRNAs and are regarded as major post‐transcriptional repressors of gene expression by directly binding to target messenger RNAs (mRNAs) and decreasing transcript stability and/or translation (Shang et al. 2023). Our previous work has demonstrated the involvement of several intestinal epithelial microRNAs in mucosal growth, adaptation, and differentiation (Kwon et al. 2021; Zhuang et al. 2013) through these mechanisms. Recently, a microRNA atlas across all small intestinal epithelial lineages was defined by multi‐omic analysis and revealed a unique expression pattern of microRNAs in specified IECs (Shanahan et al. 2021). Several microRNAs, including miR‐152, are identified as Paneth cell enriched markers through single cell sequencing, but their biological roles in intestinal epithelial defense and Paneth cells remain largely unknown.

In this study, we demonstrate evidence of impaired Paneth cell function and increased mucosal vulnerability in small intestines of aging humans and mice. The Paneth cells of aging guts exhibit significant deteriorations of mitochondrial morphology and metabolism. Our mechanistic investigation reveals a specific elevation of Paneth cell enriched miR‐152 with age. The increased levels of miR‐152 repress PHB1 expression through direct interaction with Phb1 mRNA, inhibit mitochondrial activity, and lead to Paneth cell dysfunction. In addition, our study suggests that the inhibitory role of miR‐152 in Paneth cells is fine‐tuned by intestinal intrinsic factor *circHIPK3*. However, this modulation dissipates with age due to diminished *circHIPK3* expression and decoy action. These findings suggest that dysregulated miR‐152 activity plays a central role in disordered Paneth cell metabolism and function in aging small intestine, providing translational insights for older patients with Paneth cell defects and increased intestinal mucosal vulnerability to pathological stresses.

## 2 Results

### 2.1 Increased Small Intestinal Vulnerability and Impaired Paneth Cell Function in Older Surgical Patients

**FIGURE 1:**
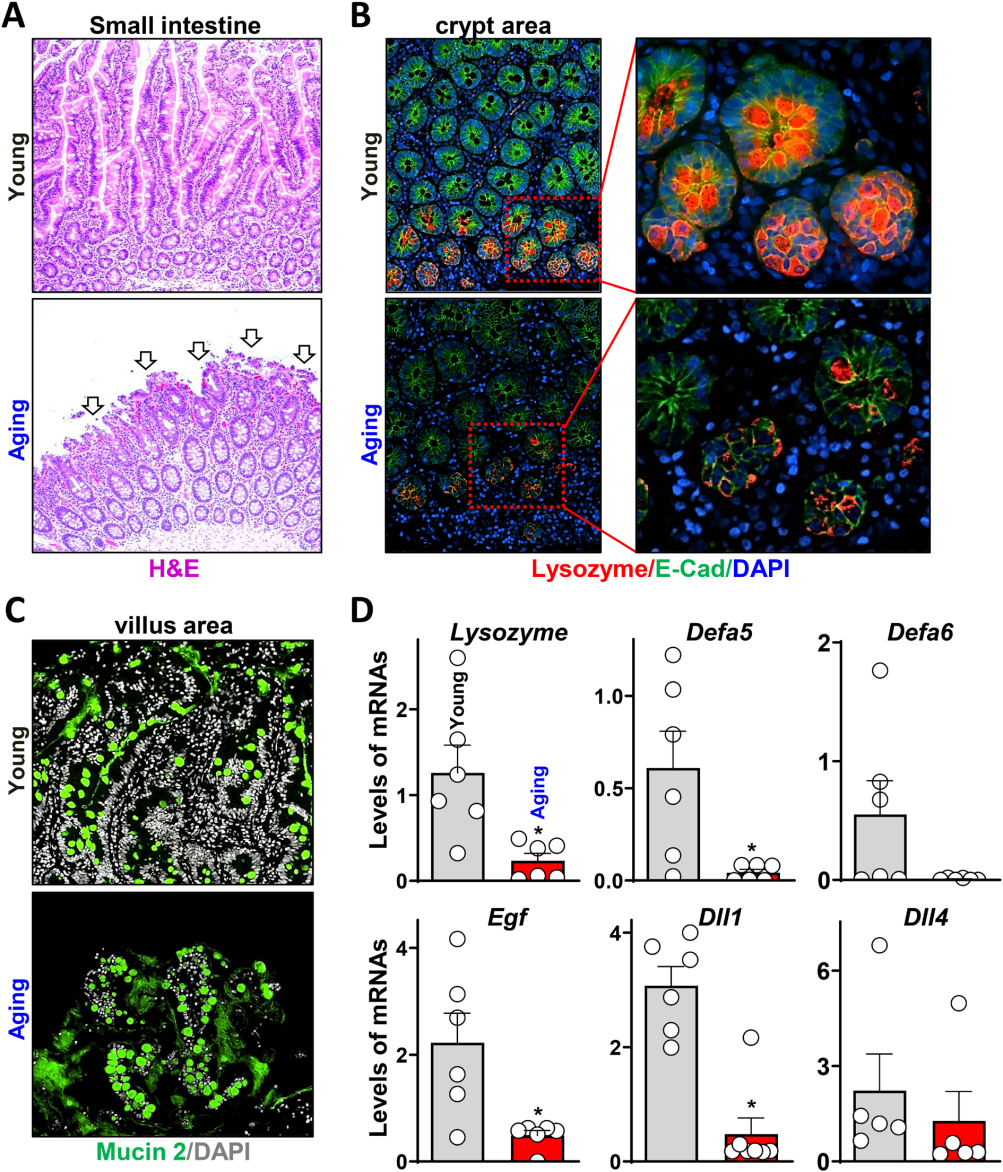
Small intestines of older patients exhibit increased mucosal vulnerability and decreased Paneth cell function. (A) H&E staining of small intestines from young (≤ 35 years old) and aging (≥ 60 years old) individuals under surgical stresses. (B) Paneth cells marked by Lysozyme staining in the crypt area of small intestines described in (A). (C) Goblet cells marked by Mucin2 staining in the villus area of small intestines described in (A). (D) Expression levels of Panth cell released factors by Q‐PCR from the individuals described in (A). Values are means ± SEM (*n* = 6). *p < 0.05 compared with young group.

To observe how aging affects small intestinal vulnerability and Paneth cell function in older individuals, we examined small intestine tissues resected from surgical patients who suffered abdominal injury and underwent emergent surgery without any preexisting chronic intestinal diseases. As shown in Figure 1A with a longitudinal view, the structural continuity of small intestines was well preserved in younger surgical patients (≤ 35 years old, Young), but there were markedly elevated mucosal damages of truncated and broken villi with micro bleeding (arrowed) across the entire mucosa in older patients (≥ 60 years old, Aging) under similar critical conditions. To assess the Paneth cell function, Lysozyme, one of the most abundant AMP produced by Paneth cells, was used for tissue immunofluorescence to monitor their secretory activity in the small intestinal crypt area (Figure 1B). Compared with the Paneth cells of the young cohort with normal package efficiency, the intensity of Lysozyme signal and the number of Lysozyme positive Paneth cells were significantly reduced in the crypts of aging small intestines. In addition, we examined the activity of another secretory IECs, Goblet cells, by assessing their mucin2 production. As shown in Figure 1C and Figure S1A, mucin2 positive Goblet cells were not reduced in number or intensity in the small intestines. There was neither a reduction in Goblet cell activity nor severe structural disruption in the colons of the aging individuals (Figure S1B,C), indicating a selective impact of aging on small intestinal Paneth cells.

As introduced previously, Paneth cells play two essential roles in the crypts: they function as innate immune cells by secreting antimicrobial molecule AMPS; and niche cells by providing essential paracrine signals to adjacent stem cells. Therefore, we further confirmed the functional changes of Paneth cells with age by examining the gene expressions of several AMPs and niche factors specifically released by human Paneth cells, including mRNAs of Lysozyme, Defa5, Defa6, Dll1, Dll4, and Egf. As shown in Figure 1D, Lysozyme, Defa5, Egf, and Dll1 were significantly reduced in small intestines of aging individuals compared to young, but no significant reduction was found in Defa6 or Dll4 levels. Taken together, our results provided unambiguous evidence that aging specifically and profoundly impairs secretory function of human Paneth cells, along with decreased small intestinal capability to maintain structural and functional integrity under critical surgical stress.

### 2.2 Paneth Cell Dysfunction and Increased Vulnerability in Aging Human Organoids

**FIGURE 2:**
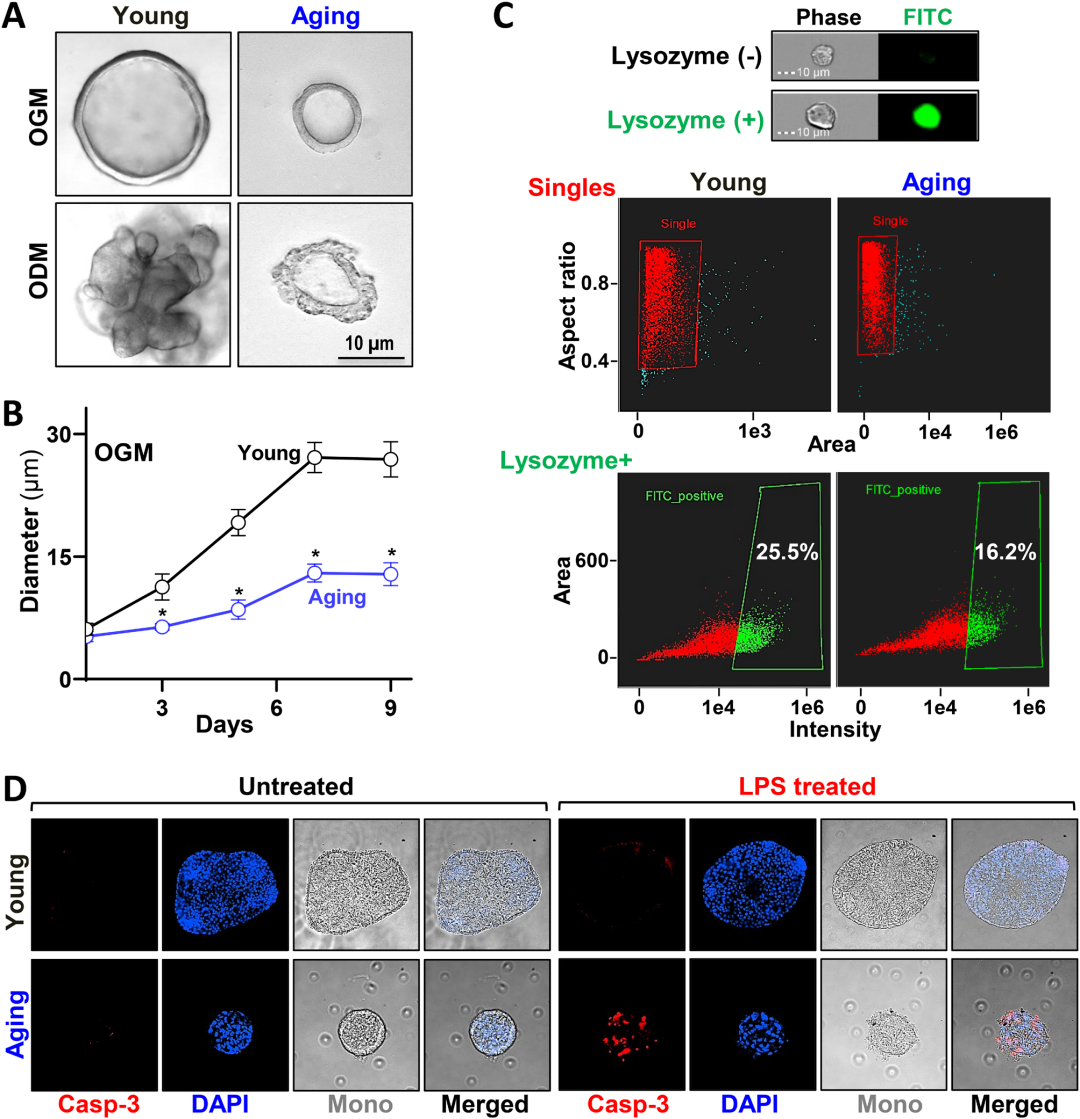
Human small intestinal organoids demonstrate age‐associated decrease in Paneth cell function and increase in vulnerability to stress. (A) Images of young and aging organoids maintained in growth (OGM) and differentiation (ODM) culture medium, on day 7 and day 14 respectively. (B) Measurement of organoids cultured in OGM described in (A). Values are the means ± SEM (*n* = 10). *p < 0.05 compared with young group. (C) Image flow cytometric analysis of Lysozyme positive cell population in organoids described in (A). *n* > 5000 cells/group. (D) Cleaved caspase‐3 staining in organoids with or without LPS treatment. Experiments were repeated 3 times and showed similar results.

To corroborate the in vivo observations of age‐associated Paneth cell dysfunction in human small intestines, we used a more simplified but physiologically relevant ex vivo model: small intestinal organoids derived from healthy young (≤ 35 years old) and aging (≥ 60 years old) donors. As shown in Figure 2A, both age‐grouped organoids formed typical cystic shaped colonies in organoid growth medium (OGM) and developed a more complex architecture with crypts and villi after being induced in organoid differentiation medium (ODM) for 7 days. It was noted that the aging human organoids were smaller in diameter as they grew (Figure 2B) and possessed less budding potential than the young organoids as they differentiated.

To accurately characterize the Paneth cell population in the young and aging organoids, we used Imagestream flow cytometry to capture Lysozyme positive Paneth cells from single cells prepared from the young and aging human organoids. Each captured epithelial cell was examined for structural integrity and singularity before being gated to identify the Lysozyme positive Paneth cell population (> 5000 cells/group). As shown in Figure 2C, young organoids cultured in growth medium had ~25.5% of Lysozyme positive Paneth cells, but the population decreased to ~16.2% in aging organoids. The experiment was repeated 3 times, and a consistent ~36% reduction of Paneth cells with aging was shown in the human organoid culture (Figure S2A).

Given the reduced number of Lysozyme positive Paneth cell population in the aging organoids, we hypothesized that the aging organoids would be more susceptible than young organoids to stress induced damage that resembles the in vivo findings in Figure 1A. To test this hypothesis, bacterial product lipopolysaccharide (LPS) mimicking pathogen attack was added to the culture medium of the organoids for a period of 16 h (1 μg/mL). As shown in Figure 2D and Figure S2B, no remarkable signal of cleaved caspase‐3 was detected in either young or aging organoids to indicate spontaneous cell death. Upon the LPS treatment, young organoids showed only ~5% cell death and maintained their structural integrity. However, aging organoids were severely destructed with a wrinkled edge, detachment, and marked caspase‐3 activation (~35%). Together, our ex vivo data supported the notion that small intestinal aging is associated with declined Paneth cell function and increased vulnerability to pathological stress. It also suggested that declined Paneth cell function contributes to the growth inhibition of aging small intestinal organoids given their more prominent niche role in the simplified epithelial model system (Xiao et al. 2023; Quintero and Samuelson 2025).

### 2.3 Paneth Cell Function Declines in Aging Mouse Mucosa

There are reported observations of old age‐related increase of Paneth cell population and function from studies using mice or mouse derived intestinal organoids (Nalapareddy et al. 2017; Moorefield et al. 2017), interpreted as an effort of compensation. To test whether aging impacts Paneth cells differently in older mice than human, sex matched 8‐week‐old (Young) and 76‐week‐old (Aging) C57BL/6 mice were used. Both age groups were healthy and active, though the older mice appeared to have increased depth and width of small intestinal epithelium, consistent with the intestinal hyperplasia reported by many (Figure S3Aa) (Wang et al. 2021, 2025; Hohman and Osborne 2022; Corazza et al. 1998; Ciccocioppo et al. 2002). In addition, older mice showed higher small intestinal permeability evaluated by serum FITC‐dextran (Figure S3Ab), without significant shift in the expressions of major intercellular junction proteins (Figure S3Ac). Similar to aging humans, mucin production by Goblet cells didn't decline either in small intestine (Figure S3Ad) or colon (Figure S3B) of aging mice.

**FIGURE 3:**
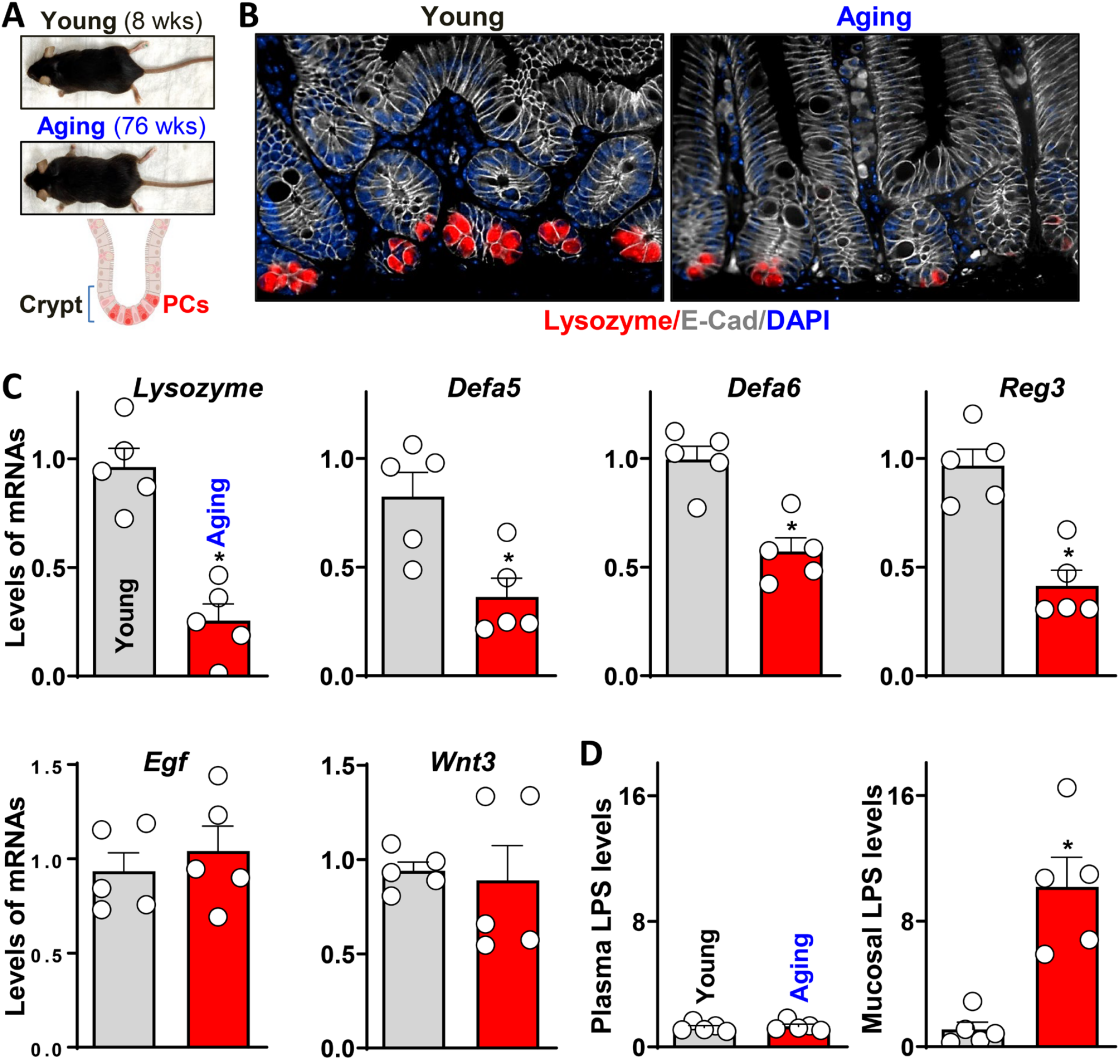
Small intestines of older mice exhibit decreased Paneth cell function. (A) Animal study design using intestines from young (8 weeks old) and aging (76 weeks old) mice. (B) Images of Lysozyme staining in the proximal small intestines of the mice described in (A). (C) Expression levels of Paneth cell released factors from the mice described in (A). Values are means ± SEM (*n* = 5). *p < 0.05 compared with young group. (D) Fold change of LPS concentration in plasma and intestinal mucosa from the mice described in (A). Values are means ± SEM (*n* = 5). *p < 0.05 compared with young group.

As shown in Figure 3A, proximal small intestines from both groups were collected to investigate Paneth cells in the crypt base. There was a notable reduction in the Lysozyme positive cell population in the aging mice compared with young mice (Figure 3B), similar to the decline shown in the older patients. We then assessed the levels of Paneth cell released factors (Nalapareddy et al. 2017; Quintero and Samuelson 2025; Bevins and Salzman 2011; Lueschow and McElroy 2020) in the small intestinal mucosa of young and aging mice. As shown in Figure 3C, in addition to the most reduction in Lysozyme, key antimicrobial factors including Defa5, Defa6, and Regenerating islet‐derived protein 3 (Reg3) were also significantly decreased in aging mouse small intestine. Consistently, there was an elevated tissue concentration of endotoxin LPS in the aging small intestinal mucosa, though the plasma LPS remained the same with age (Figure 3D). Considering the common gut dysbiosis with chronological aging, the increased tissue LPS could only be deteriorated by reduced Paneth cell defense activity. Put together, our data from young and aging mice indicates a similar and specific age‐associated decline in Paneth cell function as in human small intestine, which leads to a weakened gut barrier defense and microbial clearance capacity.

### 2.4 Aging Paneth Cells Have Mitochondrial Defects

**FIGURE 4:**
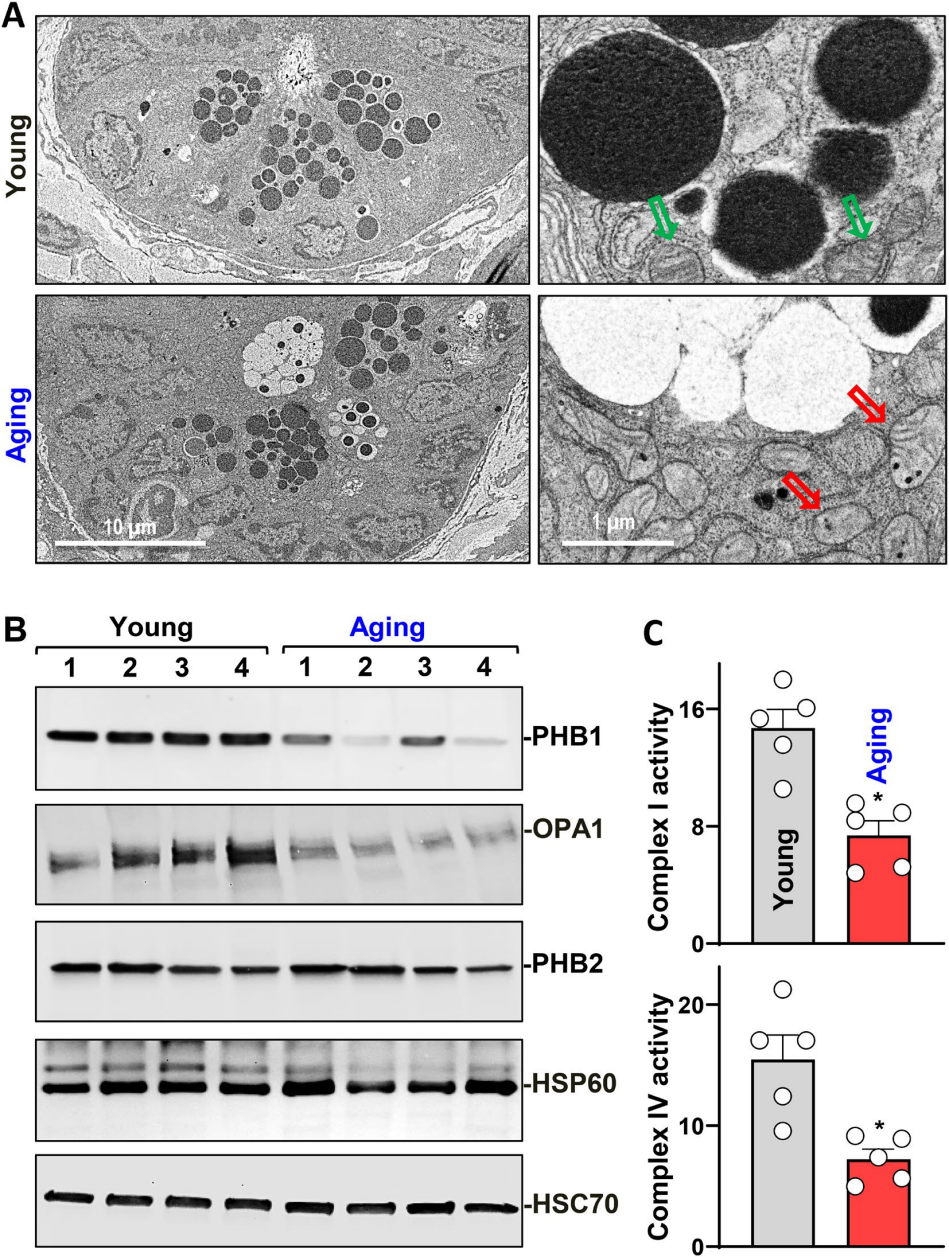
Small intestinal epithelium of older mice shows mitochondrial dysfunction. (A) EM images of small intestinal crypt from young and aging mice. Green and red arrows indicate mitochondria. (B) Immunoblots of mitochondrial proteins from small intestinal epithelium tissue extracts of young and aging mice. (C) Colorimetric absorbance of Complex I and IV activity in small intestinal epithelium of young and aging mice. Values are means ± SEM (*n* = 5). *p < 0.05 compared with young group.

To investigate the cause of the chronological decline in Paneth cell function, young and aging mouse small intestinal tissue samples were prepared and subject to electron microscopy scanning. As shown in Figure 4A, Paneth cells at the crypt base of young small intestine present normal packaged electron dense granules (top left panel), which were surrounded by healthy mitochondria of clear baseline and cristae organization (top right panel). However, Paneth cells of aging small intestine had many granules that were either electron lucent or composed of an electron dense core and an enlarged electron‐lucent peripheral halo (bottom left panel), supporting an aberrant packing and secretory function (Stappenbeck 2010; Stahl et al. 2018; Garabedian et al. 1997). Importantly, the surrounding mitochondria appeared unhealthy, swollen, with dissolution of cristae, and contained electron dense inclusion bodies (bottom right panel). The abnormal morphology of mitochondria is a key indicator of dysfunctional mitochondrial biogenesis and metabolism inside the Paneth cells of aging small intestine.

Therefore, essential mitochondrial proteins were examined in the small intestinal epithelium of young and aging mice. As shown in Figure 4B, PHB1 and Optic atrophy 1 (OPA1) were significantly decreased in the small intestinal epithelium of aging mice compared with young mice, whereas the expression of Prohibitin 2 (PHB2), Heat shock protein 60 (HSP60), Succinate dehydrogenase complex flavoprotein subunit A (SDHA), Mitofusin 1 (MFN1), and Translocase of outer mitochondrial membrane 20 (TOM20) was unchanged with age (some data not shown). In IECs, PHB1 is predominantly distributed in the mitochondria as a major component protein of the inner mitochondrial membrane (Theiss et al. 2007). PHB1 regulates OPA1 activity and plays essential roles in Complexes I and IV of the electron transport chain (ETC) (Nijtmans et al. 2000). Therefore, we examined Complex I and Complex IV activity of oxidative phosphorylation in the tissue extracts of young and aging small intestinal epithelium. As shown in Figure 4C and Figure S4, the activity of ETC Complex I and Complex IV was evidently decreased in aging samples compared with young. Together, these results strongly suggest dysfunctional mitochondrial respiration and metabolism in aging small intestine epithelium which could play a role in the age‐associated Paneth cell defects.

### 2.5 Paneth Cell Enriched miR‐152 Elevates With Age

**FIGURE 5:**
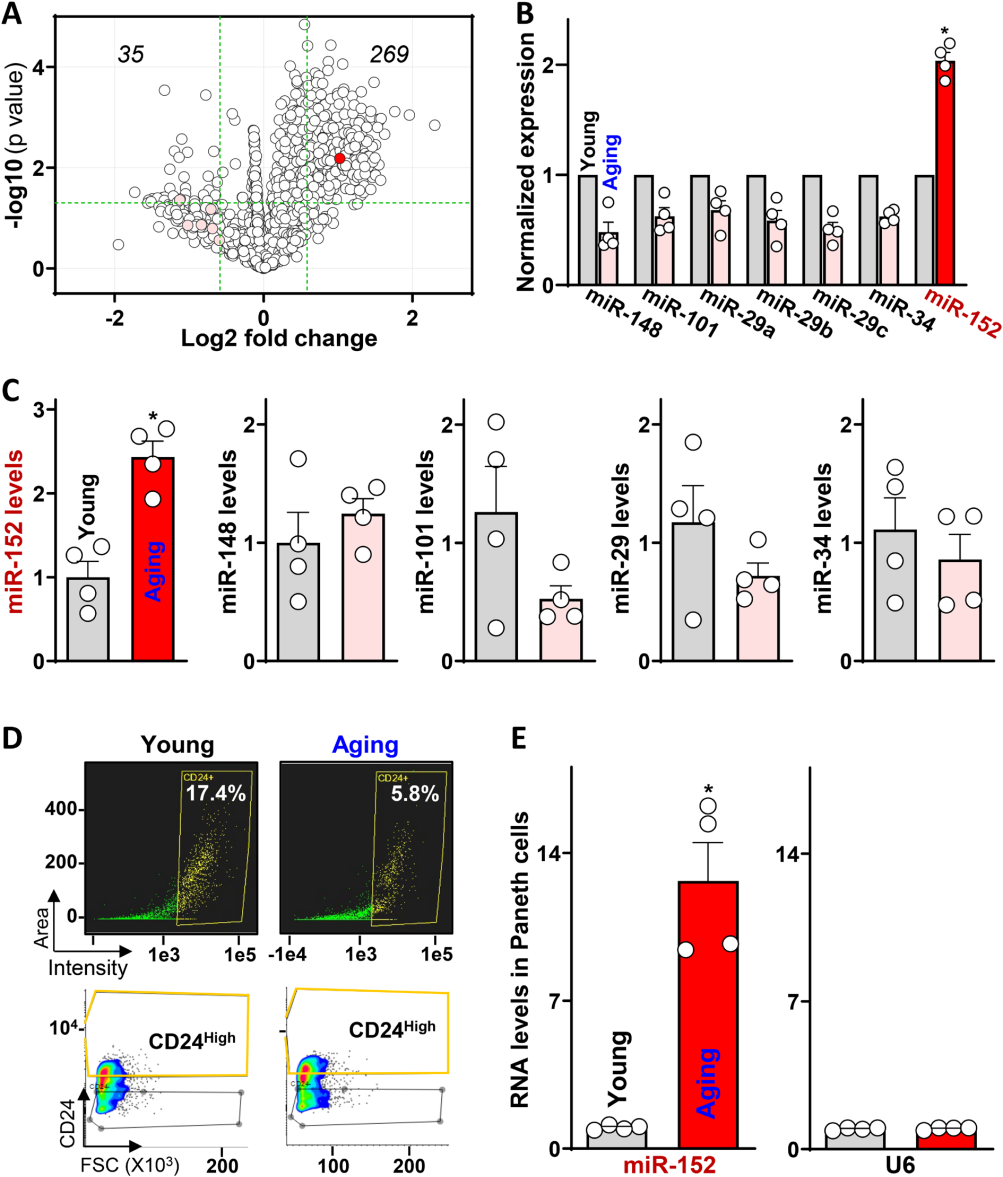
Paneth cell enriched miR‐152 increases in small intestinal epithelium of older mice. (A) MiR profiling of small intestinal epithelium of young and aging mice. (B) Relative levels of Paneth cell enriched miRs. (C) Q‐PCR of Paneth cell enriched miRs from the tissue samples described in (A). (D) Imageflow cytometric analysis and single cell sorting of CD24^High^/Lysozyme (+) cells from young and aging human small intestinal organoids. *n* > 5000 cells/group. (E) Relative miR‐152 levels in the sorted Paneth cells. Values are means ± SEM (*n* = 4). *p < 0.05 compared with young group.

To investigate the involvement of intestinal epithelial microRNAs in the age‐associated decline in mitochondrial metabolism and Paneth cell function, we profiled the microRNA expression in the small intestinal epithelium of young and aging mice and analyzed the expression of Paneth cell specific microRNAs. Consistent with previous reports that aging alters microRNA expressions in various tissues (Koch 2023; Kinser and Pincus 2020), our array data displayed a distinct microRNA profile in the aging mouse small intestinal epithelium, with 35 decreased and 249 increased microRNA expressions compared with the young (Figure 5A). However, among the Paneth cell enriched microRNAs miR29abc, miR‐34a, miR101a, miR148a, and miR‐152 (colored dots in volcano plot), miR‐152 was the only microRNA that showed a significant change with age (Figure 5B). Individual qPCR was performed to verify the expressional change of each microRNA in the epithelial tissue and the results consistently suggested a remarkable increase of Paneth cell marker miR‐152 in the aging epithelium compared with young controls (Figure 5C).

Given the specific increase of miR‐152 in aging mouse small intestine as a Paneth cell enriched microRNA, further steps were taken to define its expression in Paneth cells of young and aging human organoids. Adopting the established Paneth cell gating strategy (Goga et al. 2021; von Furstenberg et al. 2011), we collected single live CD24^high^ cells as Paneth cells from young and aging human small intestinal organoids by image flow cytometry. As shown in Figure 5D top, CD24+ cell population decreased in aging epithelial cells with an even deeper drop than Lysozyme+ population. Among CD24+ cells, the CD24^high^ cells showed an extensive overlap with Lysozyme+ cells determined by co‐staining of CD24 and Lysozyme antibodies (Figure 5D: bottom and Figure S5), which is consistent with previous reports (von Furstenberg et al. 2011; Yu et al. 2018). Therefore, the CD24^high^ cells were sorted and collected to examine the miR‐152 expression. As shown in Figure 5E, qPCR of the sorted cells demonstrated a significant increase of miR‐152 with age in human intestinal organoid Paneth cells. Taking together, our results suggest a unique increase of miR‐152 in Paneth cells of aging small intestine.

### 2.6 miR‐152 Inhibits Mitochondrial Function via Direct Control of PHB1 Expression

**FIGURE 6:**
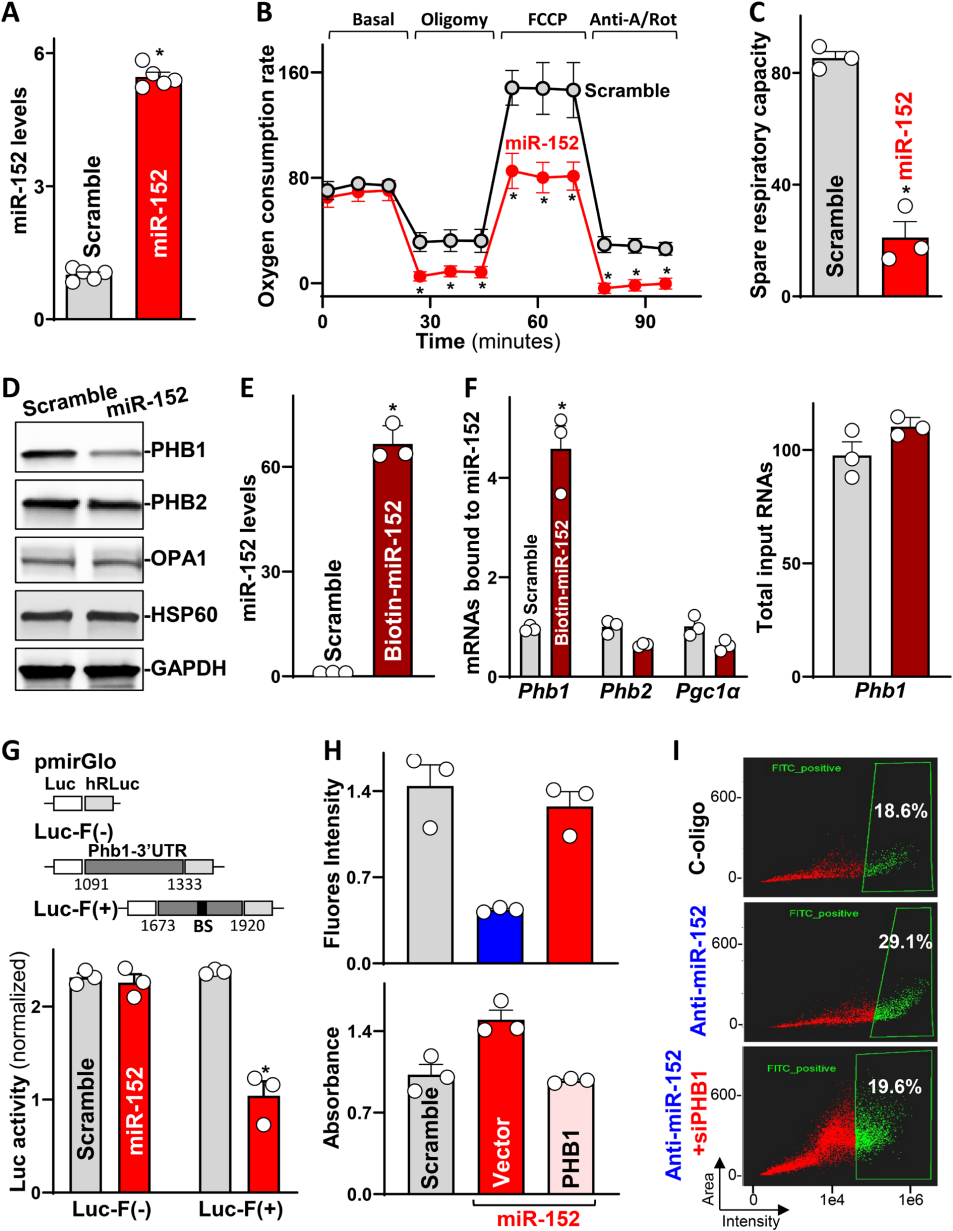
MiR‐152 disrupts mitochondrial function by inhibiting PHB1. (A) MiR‐152 overexpression in IECs. MiR‐152 mimic or scramble control was transfected in Caco‐2 cells for 48 h before miR assay to examine the levels of miR‐152. Values are means ± SEM (*n* = 5). *p < 0.05 compared with Scramble. (B) Mitochondrial respiration in cells described in (A) examined by Seahorse assay. Values are means ± SEM (*n* = 3). *p < 0.05 compared with Scramble. (C) Calculated spare respiration capacity. (D) Immunoblots of mitochondrial proteins in cells described in (A). (E) Biotin labeled miR‐152 or scrambled RNA transfection in IECs. Caco‐2 cells were transfected 48 h before qPCR to examine the levels of miR‐152. Values are means ± SEM (*n* = 3). *p < 0.05 compared with biotin labeled Scramble. (F) Pulldown assay to determine the miR‐152 bound mRNAs (left) and Phb1 mRNA levels in input RNA samples (right). Values are means ± SEM (*n* = 3). *p < 0.05 compared with biotin labeled Scramble. (G) Levels of luciferase activity of Phb1 3′UTR reporters with or without miR‐152 binding site (BS). Top, luciferase vectors; bottom, luciferase activity in IECs with or without miR‐152 overexpression. (H) Mitochondrial function assay in IECs transfected with either control, or miR‐152 mimic, or miR‐152 with PHB1 overexpression. Top, MitoTracker Green intensity; bottom, superoxide production. Values are means ± SEM (*n* = 3). *p < 0.05 compared with Scramble control. (I) Imageflow cytometric analysis of Lysozyme positive cell population in aging human intestinal organoids with transfection of control oligonucleotides, miR‐152 inhibitor, or miR‐152 and PHB1 silencer. *n* > 5000 cells/group. Experiments were repeated 3 times and showed similar results.

To investigate the potential role of miR‐152 in age‐associated Paneth cell impairment and mitochondrial decline, we began with a miR‐152 gain‐of‐function study in cultured IECs. MiR‐152 mimic was transfected in Caco‐2 cells for 48 h, which specifically elevated miR‐152 levels more than 5 folds while Scramble control RNA transfection but didn't affect other miRs such as miR‐29 and miR‐124 (Figure 6A and Figure S6A). Then the cellular mitochondrial respiration was examined with or without miR‐152 overexpression by Cell‐Mito‐Stress test on Seahorse XF extracellular flux analyzer as described previously (Xiao et al. 2023). As shown in Figure 6B,C, elevated miR‐152 expression disrupted mitochondrial homeostasis in IECs, as evidenced by reduced oxygen consumption rate (OCR) and spare respiratory capacity compared with those observed in control cells transfected with Scramble RNA. Key mitochondrial proteins including PHB1 and OPA1 were examined in the protein lysate of control and miR‐152 overexpression cells. As shown in Figure 6D and Figure S6B, PHB1 was specifically reduced in IECs overexpressing miR‐152, whereas OPA1, HSP60, SDHA, MFN1, DRP1 remained unchanged and PHB2 showed a slight decrease. Meanwhile, total mRNA levels of PHB1 were not altered by miR‐152 overexpression (Figure S6C), indicating a post‐transcriptional mechanism by miR‐152 in PHB1 gene expression. As a control, miR‐29 and miR‐124 were also transfected into the cells under the same condition as miR‐152. Though both miRs have putative binding sites to PHB1, neither altered the PHB1 expression (Figure S6D,E).

PHB1 is an evolutionarily conserved key component in mitochondria and plays indispensable roles in maintaining human Paneth cell function especially under pathological stresses (Khaloian et al. 2020; Jackson et al. 2020). Therefore, we took further steps to understand the mechanism underlying the regulatory control of miR‐152 in PHB1 gene expression and aging Paneth cells. First, we examined the interaction of miR‐152 with Phb1 mRNA by RNA pulldown assays using biotin‐labeled miR‐152, as we reported previously (Xiao et al. 2016). 48 h after biotin‐labeled miR‐152 transfection in IECs, miR‐152 levels increased significantly (Figure 6E). Phb1 mRNA was then detected enriched in the materials pulled down by transfected biotin‐miR‐152 but not from cells that had been transfected with a control biotinylated Scramble RNA (Figure 6F: left). The association of miR‐152 with Phb1 mRNA was specific because biotin‐miR‐152 did not show any interaction with Phb2 or Pgc1α mRNA. Again, the input/total Phb1 mRNA was not altered by the elevated miR‐152 levels (Figure 6F: right).

Based on the bioinformatic prediction, there is a consequential pairing region for the miR‐152 family on the 3′ untranslated region (3′UTR) of Phb1 transcript. So we constructed dual luciferase reporters with or without the 3′UTR pairing region, Luc‐F(+) and Luc‐F(−) respectively, to quantitatively evaluate the influence of miR‐152 on Phb1 gene expression (Figure 6G: top). As shown in Figure 6G bottom, increased miR‐152 expression by transfection with miR‐152 mimic selectively decreased the Luc‐F(+) luciferase reporter activity but failed to inhibit the activities of Luc‐F(−) that contain no miR‐152 binding sites. These results indicate that miR‐152 directly interacts with Phb1 mRNA via the 3′‐UTR and inhibits PHB1 gene expression.

To test if miR‐152 regulates mitochondrial function through controlling PHB1 expression, PHB1 was overexpressed in IECs to rescue the mitochondrial function when miR‐152 was simultaneously elevated. As shown by the Elisa assays in Figure 6H, mitochondrial function was impaired by miR‐152 overexpression as indicated by decreased intensity of MitoTracker Green and increased superoxide production. However, miR‐152 overexpression induced mitochondrial dysfunction was prevented by ectopically overexpressing PHB1, as the signal of MitoTracker Green and superoxide production returned to control levels in the cells co‐transfected with PHB1 overexpression vector.

To verify the influence of miR‐152 in aging Paneth cells via regulating PHB1 expression, a loss‐of‐function study with miR‐152 inhibitor was performed in aging human intestinal organoids. The endogenous levels of miR‐152 were significantly reduced by the miR‐152 inhibitor (Anti‐miR152) after 48 h transfection compared with control oligonucleotide (C‐oligo) transfection (data not shown). Then the human aging organoids were prepared for single cell flow cytometric analysis (Figure 6I). The results showed that miR‐152 inhibition remarkably increased the Lysozyme positive cell population (Figure 6I: middle) compared with control transfection (Figure 6I: top). However, the recovery of the Lysozyme positive population by miR‐152 inhibition in aging organoids was completely abolished when PHB1 was knocked down by co‐transfection of siRNA targeting PHB1 (Figure 6I: bottom). Therefore, our results suggested that Paneth cell enriched miR‐152 inhibits mitochondrial function via direct control of PHB1 expression, and age‐related increase of miR‐152 in small intestine leads to Paneth cell decline.

### 2.7 Dysregulated miR‐152 Activity Is Reinforced by the Loss of *circHIPK3* During Aging

**FIGURE 7:**
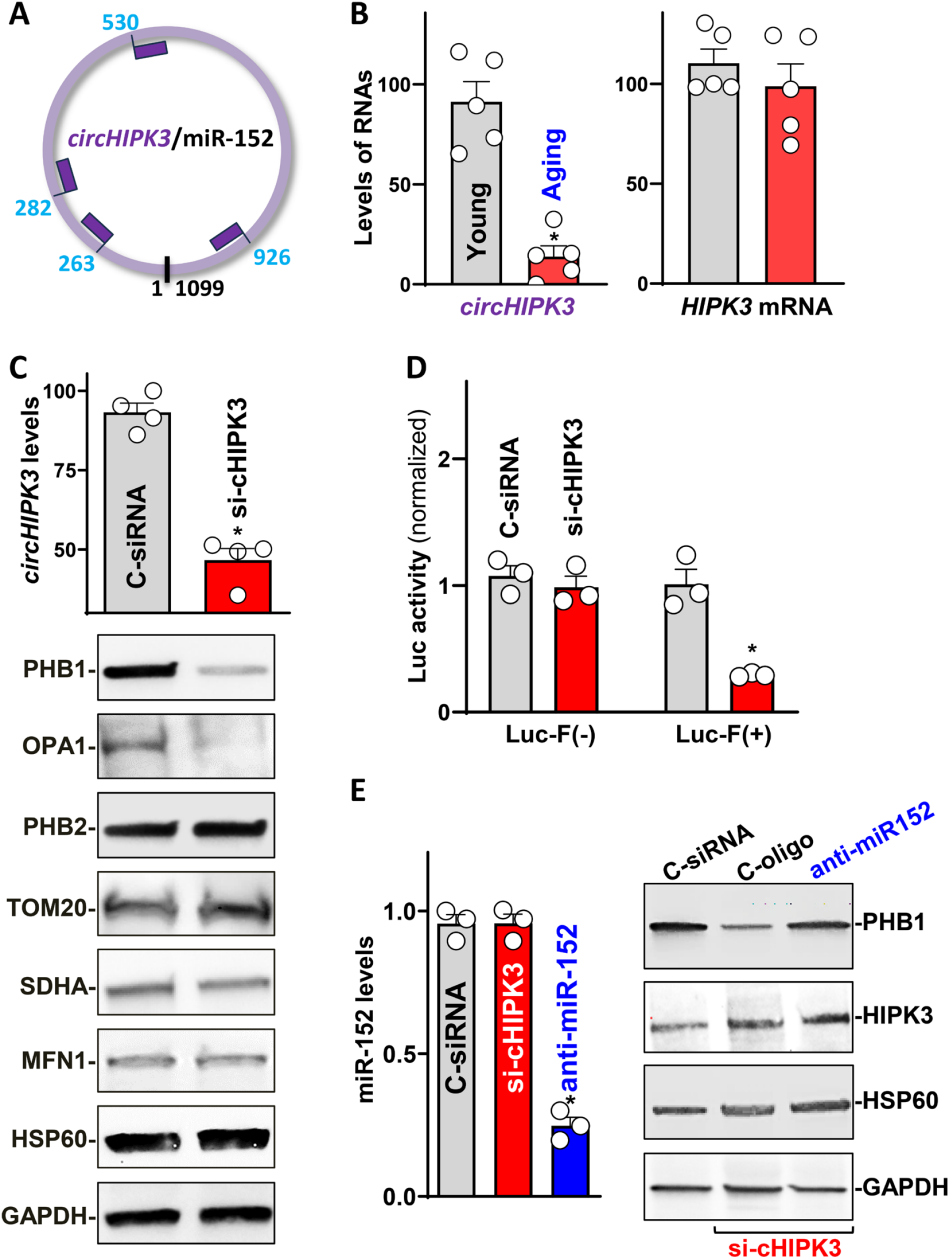
MiR‐152 activity is modulated by intestinal *circHIPK3*. (A) Schematic of putative miR‐152 binding sites on *circHIPK3*. (B) Levels of *circHIPK3* in young and aging small intestines of humans. HIPK3 mRNA served as control. Values are means ± SEM (*n* = 3). *p < 0.05 compared with young group. (C) Levels of *circHIPK3* and immunoblots of mitochondrial proteins in IECs transfected with either control siRNA or siRNA targeting the junction area of *circHIPK3* for 48 h. Values are means ± SEM (*n* = 3). *p < 0.05 compared with control siRNA. (D) Luciferase activity of Phb1 luciferase reporters after transfection with control or si‐cHIPK3. Values are means ± SEM (*n* = 3). *p < 0.05 compared with control. (E) Levels of miR‐152 and PHB1 protein in IECs 48 h after transfection with control siRNA, si‐*circHIPK3*, or si‐*circHIPK3* with miR‐152 inhibitor.

The regulatory role of any given microRNA in post‐transcriptional control of gene expression is modulated by factors such as microRNA biogenesis, intracellular localization, availability, and interplay with other trans‐acting factors. Non‐coding circular RNAs (circRNAs) are known to act as a microRNA sponge to modulate microRNA availability to target transcripts. Our previous work has demonstrated that conserved circRNA HIPK3 (*circHIPK3*) is abundantly expressed in the intestinal epithelium and protects the tissue integrity by promoting epithelial repair after injury (Xiao et al. 2021). Interestingly, *circHIPK3* carries several miR‐152 binding sites, as shown in Figure 7A. So we investigated whether *circHIPK3* is involved in the dysregulated miR‐152 activity in aging intestinal epithelium. First, we tested and compared *circHIPK3* expressions in human small intestines of young and aging individuals with convergent primers that specifically amplify the junction area of the circRNA (Figure S7A). Contrary to the increased expression of miR‐152 during aging, *circHIPK3* levels decreased in older human small intestines compared with the young (Figure 7B), whereas the host gene HIPK3 mRNA or other circular RNA circZNF609 did not show any change (Figure S7B). Then, we explored the effect of lost *circHIPK3* on mitochondrial protein expressions in cultured IECs. As shown in Figure 7C and Figure S7C, 48 h after transfection of siRNA specifically targeting the junction area of *circHIPK3*, PHB1 was significantly reduced, which is similar to the inhibitory effect of miR‐152. OPA1 was also slightly reduced, whereas other key mitochondrial proteins remained unchanged. In addition, the oxygen consumption rate tested by Seahorse assay was significantly less in IECs with *circHIPK3* knockdown (Figure S7D).

Finally, we tested whether loss of *circHIPK3* dampens mitochondrial function due to the diminished decoy to miR‐152. Again, dual luciferase PHB1 reporters with or without miR‐152 binding site were transfected in IECs with *circHIPK3* knockdown. As shown in Figure 7D, luciferase activity decreased in *circHIPK3*‐silencing cells when transfected with the Luc‐F(+) reporter, primarily resulting from increased availability of miR‐152. Luciferase activity was not affected by *circHIPK3* knockdown when cells were transfected with the null vector or Luc‐F(−) reporter. These results suggest that *circHIPK3* sequesters/inhibits miR‐152 activity in cultured IECs, and loss of *circHIPK3* enhances miR‐152 inhibitory activity. To confirm the functional consequence of reduced *circHIPK3* decoy to miR‐152 activity, PHB1 expression was examined in the same condition. As shown in Figure 7E, *circHIPK3* knockdown alone by siRNA transfection didn't change miR‐152 content, but it robustly decreased the levels of cellular PHB1 expression. Interestingly, *circHIPK3* knockdown‐induced inhibition of PHB1 expression was prevented by decreasing miR‐152 through co‐transfection with miR‐152 inhibitor. The levels of PHB1 expression in cells co‐transfected with *circHIPK3* siRNA and miR‐152 inhibitor were similar to those observed in cells transfected with control. Together, these results indicate that the inhibitory role of miR‐152 in PHB1 expression in IECs is modulated by *circHIPK3* sponge effect. Therefore, age‐associated decrease of *circHIPK3* expression enhances miR‐152 activity to deteriorate mitochondrial function.

## 3 Discussion

Age‐associated changes in the gut, especially the gut barrier and defense function, not only are involved in an array of chronic disorders but also increase the risks for infection and severe complications in older surgical patients. However, our current knowledge of how age adversely impacts the functionality of the intestinal barrier and the mechanisms that underlie the decreased mucosal defense remains limited. This work represents our first step towards a better understanding of both. We provided direct evidence from aging human and mouse small intestines to demonstrate that Paneth cells, as the most essential players in epithelial defense, are impaired during aging with significant mitochondrial dysfunction. Our mechanistic investigation revealed that Paneth cell enriched miR‐152 is markedly increased in aging small intestinal epithelium. Elevated miR‐152 disrupts mitochondrial activity by directly interacting with Phb1 3′UTR and inhibiting PHB1 gene expression. Contrarily, antagonizing endogenous miR‐152 in aging intestinal organoids enhances Paneth cell function through restored PHB1 expression. In addition, we found that the old age‐associated miR‐152 activation is reinforced by decreased *circHIPK3* decoy. These findings indicate that dysregulated miR‐152 activity plays a central role in disordered Paneth cell metabolism and function in the aging small intestine. The newly discovered path of *circHIPK3*/miR152/PHB1 that leads to age‐associated Panth cell impairment provides novel translational insight for older patients with weakened intestinal mucosal defense and Paneth cell defects.

Gut is always considered a key mediating center for the aging process, but our actual knowledge regarding the functional changes of gut with age is extremely limited and actively debated (DeJong et al. 2020; Wilms et al. 2020; Mabbott 2015; Valentini et al. 2014). As the small intestine exclusive epithelial cells, Paneth cells have drawn heavy attention due to their unique features and irreplaceable roles in maintaining gut homeostasis (Quintero and Samuelson 2025). Some studies suggested an increased Paneth cell population in aged mouse intestine (Nalapareddy et al. 2017, 2022; Moorefield et al. 2017), whereas human study on this subject has always been inconclusive. Our work directly addressed this important aging question in human and mouse small intestines with approaches including AMP staining, quantitative PCR of Paneth cell released factors, single cell image flow cytometry, and scanning electron microscope (SEM) to assess various aspects of Paneth cell functionality. All the results consistently pointed to the notion that Paneth cells decline with chronological aging. Consequentially, impaired Paneth cell function leads to increased vulnerability in aging gut mucosa, as indicated by elevated local endotoxin concentration, enhanced apoptotic death upon LPS treatment, and more severe tissue damage under stressful conditions such as trauma and surgical intervention.

Interestingly, our study suggested that the impact of aging is not universally placed on the other key barrier defender epithelial cells, the mucin‐producing Goblet cells. Different than Paneth cells, Goblet cells populate in both small intestinal and colonic epithelium. Highly O‐glycosylated mucin produced by Goblet cells works hand in hand with Paneth cell secreted AMPs to prevent microbial invasion in small intestines. In colon where Paneth cells are absent, the thick mucus layer formed by Goblet cells is the major force of the mucosal innate immune system. Our tissue staining results in small intestine and colon tissues suggest that Goblet cell function is not altered during aging either in humans or mice, as the mucin production remained the same. Additionally, our limited study with human colon tissues demonstrated that the structural integrity of the colon is well maintained in aging patients under stressful surgical conditions. These results indicate less involvement of Goblet cells in age‐associated increase of intestinal vulnerability and emphasize an irreplaceable role of Paneth cells in protecting intestinal integrity during aging.

Our study also provided novel evidence of mitochondrial dysfunction in Paneth cells of aging small intestines. Mitochondrial dysfunction was obvious with morphological abnormity within the Paneth cells, altered mitochondrial protein expressions, and diminished activity of Complexes I and IV of the electron transport chain. It is noticed that both PHB1 and OPA1 were significantly reduced in aging small intestinal epithelium. Loss of PHB1 is known to drive mitochondrial dysfunction in Paneth cells, which is regarded as central to inflammatory bowel diseases (Khaloian et al. 2020; Jackson et al. 2020). However, OPA1 also plays a direct role in maintaining mitochondrial function by maintaining cristae junction and cristae tightness (Lee et al. 2023). So this age‐associated decrease in OPA1 levels could also contribute to the observed mitochondrial dysfunction. We have not yet thoroughly investigated the mechanism underlying the altered OPA1 expression with age. However, we suspect that the reduced OPA1 could result from the altered PHB1, since PHB1/2 complex protects and regulates OPA1 as a membrane‐associated chaperone/holdase (Nijtmans et al. 2000).

Our mechanistic study illustrated the novel biological role of Paneth cell specific miR‐152 in regulating mitochondrial metabolism. MiR‐152 was previously recognized as a Paneth cell enriched miRNA through multi‐omics analysis in small intestine (Shanahan et al. 2021), but the exact function carried out by miR‐152 in Paneth cells was not investigated. Our data revealed that miR‐152 reduces oxygen consumption rate and spare respiratory capacity in cultured IECs. Among all the key mitochondrial proteins tested, miR‐152 specifically inhibits PHB1 protein levels without affecting the total mRNAs. The RNA affinity assay confirmed the selective interaction between miR‐152 and Phb1 mRNA. The dual luciferase assay further suggested that miR‐152 modulates Phb1 translation via the seed region on Phb1 mRNA 3′UTR. Given the significant role of miR‐152 revealed here in the aging Paneth cells, it would be interesting to investigate the regulation of miR‐152 biogenesis during aging in our future study.

The findings from our current work provided potential pathways to modify and improve Paneth cell function in aging small intestines. First, the Lysozyme positive Paneth cell population in aging intestinal organoids was largely reversed by antagonizing endogenous miR‐152 with single‐stranded oligonucleotides. It would be interesting to introduce Paneth cell specific miR‐152 knockdown in an aging mouse model to investigate the potential local and systemic benefits from improved Paneth cell function. Second, ectopic PHB1 expression in cultured IECs where miR‐152 was artificially increased to mimic the age‐associated change recovered mitochondrial function, whereas PHB1 knockdown in aging intestinal organoids abolished the beneficial effect from miR‐152 inhibition. Therefore, PHB1 could serve as an ideal target in our future study to repair Paneth cell function of old intestines. Last but not least, *circHIPK3* was approved to be an efficient sponge to miR‐152 and modulate PHB1 expression via reducing miR‐152 availability to Phb1 mRNA. Restoring *circHIPK3* levels in old intestine would also be a potential path to improve aging Panth cell function. In summary, these findings suggest that *circHIPK3*/miR‐152/PHB1 axis is a potential therapeutic target to enhance Paneth cell function and improve intestinal mucosal defense in older patients.

## 4 Methods

### 4.1 Studies in Murine and Human Tissues

Both male and female mice of C57BL/6 background were purchased from The Jackson Laboratory. 8 weeks old mice served as young control while 76 weeks old mice served as aging objects. All mice were housed and cared for by trained technicians and veterinarians. Two portions of each intestine fragment (proximal small intestines) from the same location were taken, one for histological examination and the other for extraction of protein and RNA. Whole tissues were fixed in formalin and paraffin for immunohistochemical staining, whereas the mucosa was scraped with a glass slide for various measurements, as described previously (Xiao et al. 2018; Yu et al. 2011). All animal experiments were performed in accordance with NIH guidelines and were approved by the Institutional Animal Care and Use Committee of University of Maryland School of Medicine. Human tissue samples were obtained from surplus discarded tissue from the University of Maryland Medical Center. Tissues samples were examined by a pathologist and surgeon before stored in liquid nitrogen until they were assayed. Dissected and opened intestines were mounted onto a solid surface and fixed in formalin and paraffin. The study was approved by the Institutional Review Boards of University Maryland.

### 4.2 Cell and Intestinal Organoid Culture

Human colorectal carcinoma Caco‐2 cells were purchased from American Type Culture Collection and maintained under standard culture conditions (Yu et al. 2018). The culture medium and fetal bovine serum were purchased from Invitrogen, and biochemical reagents were from Sigma‐Aldrich. Isolation and culture of primary mouse enterocytes were conducted following the method described previously (Yu et al. 2020, 2022). Briefly, primary crypts were released from the small intestinal mucosa of mice; isolated crypts were mixed with Matrigel (Corning 356231) and cultured in mouse IntestiCult organoid growth medium (Stemcell Technologies 06005). The growth of organoids was examined by measuring organoid cross‐sections using the NIS‐Elements AR5.30.02 program. Human small intestinal organoids were purchased from Sigma (SCC322, SCC324, SCC326, SCC320) and maintained with IntestiCult Organoid Growth (06010) or Differentiation Medium (100‐0214) from Stemcell Technologies.

### 4.3 Imagestream Flow Cytometry and FACS Analysis

Single cells from cultured organoids were prepared with Gentle cell dissociation buffer (Stemcell Technologies 100‐0485). After incubation with antibodies specifically for flow cytometry (Table 1) at vendor recommended concentration and time, cells were washed with PBS/1% BSA and analyzed in Amnis ImageStream Imaging flow cytometer (Cytek) with MultiMag 60X objective lens to capture properly stained cells for each group (≥ 5000 cells/sample). The data from single cells were validated and analyzed on IDEAs. Live cells were isolated and labeled with CD24‐PB antibody and subjected to FACS. The top CD24 positive cells were designated CD24^High^ and were gated for collection.

**TABLE 1:** Antibodies for flow cytometry.

Lysozyme	Abcam # ab270648
DCLK1	Invitrogen # 68234‐1‐IG
CD24	Invitrogen # 17‐0247‐41
APC‐IgG	Invitrogen # 17‐4714‐81
DAPI	Sigma # D9542‐1MG

### 4.4 Q‐PCR Analysis

Total RNA was isolated using the RNeasy mini kit (Qiagen). Reverse transcription and PCR amplification reactions were performed as described (Cairns et al. 2025). The levels of Gapdh were examined to monitor RNA input in RT‐PCR samples. Real‐time quantitative PCR (Q‐PCR) analysis was conducted using StepOne Plus Systems with specific primers, probes, and software (Applied Biosystems). For miRNA studies, miR‐152 levels were quantified by Q‐PCR using the Taqman MicroRNA assay; small nuclear RNA U6 was used as an endogenous control. All primers used for Q‐PCR analysis were purchased from Thermo Fisher Scientific (Table 2).

**TABLE 2:** TaqMan probes for qPCR.

Lysozyme (hu lyz)	Invitrogen # Hs00426232_m1
Lysozyme (mm lyz1)	Invitrogen # Mm00657323_m1
Defa5	Invitrogen # Hs00360716_m1
Defa6	Invitrogen # Hs00427001_m1
DLL1	Invitrogen # Hs00194509_m1
DLL4	Invitrogen # Hs00184092_m1
REG3g (hu)	Invitrogen # Hs01595406_g1
REG3g (mm)	Invitrogen # Mm00441127_m1
EGF	Invitrogen # Hs01099990_m1
TGFα	Invitrogen # Mm00446232_m1
WNT3	Invitrogen # Mm00437336_m1
WNT6	Invitrogen # Mm00437353_m1
WNT9	Invitrogen # Mm00457102_m1
WNT11	Invitrogen # Mm00437327_g1
NRG1	Invitrogen # Mm01212130_m1
EREG	Invitrogen # Hs00914313_m1
GAPDH (hu)	Invitrogen # 4326317E
GAPDH (mm)	Invitrogen # 4352339E
miR‐152	Invitrogen # 4427975 000475
miR‐29	Invitrogen # 4427975 000413
miR‐34a	Invitrogen # 4427975 000426
miR‐101	Invitrogen # 4427975 002253
miR‐148	Invitrogen # 4427975 000470
U6 snRNA	Invitrogen # 4427975 001973

### 4.5 Immunoblotting Analysis

Whole‐cell lysates were prepared using 2% SDS, sonicated, and centrifuged at 4°C for 15 min, as described (Sharma et al. 2025). The supernatants were boiled and size‐fractionated by SDS‐PAGE. Antibody information was detailed in Table 3. After the blots were incubated with primary antibody and then secondary antibodies, immunocomplexes were developed by using chemiluminescence. Relative protein levels were analyzed by using Biorad Chemidoc and XRS system equipped with Image lab software (version 4.1).

**TABLE 3:** Antibodies for immunoblotting analysis.

PHB1	Cell Signaling Technology # 2426S
PHB2	Cell Signaling Technology # 14085S
OPA1	Cell Signaling Technology # 80471S
HSP60	Cell Signaling Technology # 12165S
HSP70	Enzo Life Sciences # ADI‐SPA‐815
ZO1	Cell Signaling Technology # 5406S
JAM1	Invitrogen #36‐1700
Occludin	Cell Signaling Technology # 91131S
Claudin‐1	Cell Signaling Technology # 4933S
SDHA	Cell Signaling Technology # 11998S
Mitofusin	Cell Signaling Technology # 14739S
DRP1	Cell Signaling Technology # 8570S
GAPDH	Cell Signaling Technology # 2118S
Anti‐rabbit IgG	Cell Signaling Technology # 7074S
Anti‐mouse IgG	Cell Signaling Technology # 7076S

### 4.6 Seahorse Metabolic Assays

Seahorse Bioscience XFe24 Analyzer was used to measure mitochondrial respiratory capacity in cultured IECs (Cairns et al. 2025). Cells were grown on a 24‐well XFe96 plate at a cell density of 20,000 cells/well. Cartridge plates for metabolic stress injections were hydrated for at least 12 h at 37°C prior to the assay with calibrant solution. 1 h before running the seahorse assay, the cell culture medium was removed and replaced with Seahorse Assay Medium (Agilent). The following compounds (final concentrations) were sequentially injected into each well: oligomycin (1.5 μM), FCCP (0.25 μM), and rotenone/antimycin (0.5 μM). Oxygen consumption rate was measured under basal conditions and after each injection using an XFe24 extracellular flux analyzer (Seahorse Bioscience). Key parameters of mitochondrial function were calculated and analyzed on Wave (Agilent). Mitochondrial activity and intracellular superoxide production were examined by using Elisa kits MitoTracker Green and MitoSox kits (Invitrogen M7514 and M7512) and performed according to the manufacture's instruction, as described (Xiao et al. 2023). Complex I (Abcam109721) and IV (Abcam109911) enzyme activity of mouse tissues were examined by using colorimetric kits from Abcam.

### 4.7 Immunofluorescence Staining and SEM

The immunofluorescence staining procedure of intestinal mucosal tissues and organoids was carried out according to the method described previously (Xiao, Li, et al. 2019; Xiao et al. 2026). Slides were fixed in 3.7% formaldehyde in phosphate‐buffered saline and rehydrated. All slides were incubated with the primary antibodies (Table 4) in blocking buffer at a concentration of 1:200 dilution at 4°C overnight and then incubated with a secondary antibody conjugated with Alexa Fluor for 2 h at room temperature. After rinsing 3 times, some slides were incubated with 1 μM DAPI (Electron Microscopy Sciences 17895) for 10 min to stain cell nuclei. Finally, the slides were washed, mounted, and viewed through a Nikon Eclipse Ti microscope. Slides were examined in a blinded fashion and decoded only after examination was completed. Images were processed and analyzed using software Nikon NIS‐Elements AR5.30.05. For electron microscopy, fresh mouse small intestines were cut into small pieces and placed into 2% paraformaldehyde and 2.5% glutaraldehyde in 0.1 M sodium cacodylate buffer for 1 h. Samples were then incubated in 1% osmium tetroxide for 2 h, followed by 1% uranyl acetate overnight at 4°C. Then samples were dehydrated through an ethanol series and embedded in Embed812 resin. After polymerization at 60°C for 48 h, 200 nm thin sections of resin were placed onto silicon wafer chips. Imaging was performed in an Apreo 2S LV FEG Scanning Electron Microscope (Thermo Fisher) using the backscatter detector and inverting the contrast for a TEM‐like image.

**TABLE 4:** Antibodies for immunofluorescence staining.

Lysozyme	Invitrogen # PA5‐89275
E‐Cadherin	BD Biosciences # 610182
MUC2	Abcam # ab272692
Ki67	Abcam # ab15580
DAPI	Cell Signaling Technology # 4083S
Anti‐Rabbit IgG (H + L)	Invitrogen # A32731
Anti‐Mouse IgG (H + L)	Invitrogen # A32727

### 4.8 Plasmid Construction

The fragments of Phb1 3′‐UTR were subcloned into the pmirGLO Dual‐Luciferase miRNA Target Expression Vector (Promega) to generate the pmirGLO‐Luc‐Phb1+/− reporters constructs as described (Zhuang et al. 2013; Xiao, Gorospe, and Wang 2019). The primer sequences for generating these constructs are provided in Table 5. Transient transfections in Caco‐2 cells were performed using the Lipofectamine Reagent as recommended by the manufacturer, and the levels of firefly luciferase activity were normalized to Renilla luciferase activity.

**TABLE 5:** Oligos for plasmid construction.

Luc‐Phb1‐3′UTR‐(+) FW	GAG CTC GAC CGA GAT GTG AGT CCT GT
Luc‐Phb1‐3′UTR‐(+) RV	TCT AGA GGA AGG TCT GGG TGT CAT TTA T
Luc‐Phb1‐3′UTR‐(−) FW	GAG CTC CAT GAT TGG CTT AAA GTG AAG GA
Luc‐Phb1‐3′UTR‐(−) RV	TCT AGA GCT CCC CAG TCC ATC ACA TA

### 4.9 Biotin‐Labeled miR‐152 Pull‐Down Assays

Biotinylated‐RNA pull‐down assays were conducted as described previously (Kwon et al. 2021). After biotin‐labeled miR‐152 was incubated with cytoplasmic lysate at room temperature for 1 h, the mixture was mixed with Streptavidin‐Dynal beads (Invitrogen) and incubated at 4°C on a rotator overnight. The beads were washed thoroughly, and the beads‐bound RNA was isolated and subjected to RT followed by Q‐PCR analysis.

### 4.10 Statistical Analysis

All values were expressed as the means ± SEM. Unpaired, two‐tailed Student's *t*‐test was used when indicated with *p* < 0.05 considered significant. When assessing multiple groups, one‐way ANOVA was utilized with Tukey's post hoc test. The statistical software used was GraphPad Instat Prism 10. For non‐parametric analysis rank comparison, the Kruskal–Wallis test was conducted.

## Author Contributions

Bridgette Warner, Haonan Zhao, and Hongxia Chen performed most experiments and summarized data. Amy VanderStoep, Ana S.G. Cuningham, Dongyoon Yoo, and Hee K. Chung participated experiments in vivo, immunoprecipitation assays, and experiments conducted in intestinal organoids and cultured IECs. Christine Brantner provided expertise in tissue Electron Microscopy. Jennifer Coleman participated in human sample collection and characterization. Joungil Choi provided expertise in Image flow cytometry. Rosemary Kozar provided expertise in patient sample selection and preparation. Myriam Gorospe participated in date analysis and edited the manuscript. Jian‐Ying Wang and Lan Xiao designed experiments, analyzed data, prepared figures, and drafted the manuscript. All authors reviewed the final manuscript.

## Funding

This work was supported by grants from the National Institutes of Health (NIH) (R01AG084613, DK57819, DK61972, DK68491, S10OD030274); and Merit Review Award from the US Department of Veterans Affairs. This work was also supported by the University of Maryland School of Medicine's and School of Dentistry's Electron Microscopy Core Imaging Facility (EMCIF).

## Conflicts of Interest

Jian‐Ying Wang is a Senior Research Career Scientist at the Biomedical Laboratory Research and Development Service (US Department of Veterans Affairs). The other authors declare no conflicts of interest.

## Supporting information


**Figure S1:** (A) Goblet cell population in small intestines from individuals described in Figure 1. (B) H&E and Mucin2 staining in colons from same individuals described in Figure 1. (C) Goblet cell population in colons from individuals described in Figure 1.
**Figure S2:** (A) Lysozyme positive cells sorted by Imagestream flow cytometry. The organoids were maintained either in growth medium (OGM) or differentiation medium (ODM) as described in Figure 2C. The experiment was repeated 3 times. (B) Percentile of Caspase3 positive cells in the organoids described in Figure 2D. The Values are the means ± SEM (*n* = 3).
**Figure S3:** (A) Small intestinal (a) H&E staining and measurements (b) permeability assay with FITC‐dextran (c) intercellular junction protein expressions (d) mucin2 positive cell population in young (8‐wks) and aging (76‐wks) mice quantified as mucin2+ cells per 100 cells (DAPI+) in crypt‐villus units. (B) Colonic (a) H&E staining (b) mucin2 staining (c) mucin2 positive cell population in mice described in (A).
**Figure S4:** Time course study of colorimetric absorbance of (A) Complex I and (B) Complex IV activity in small intestinal epithelium of young and aging mice. Values are the means ± SEM (*n* = 5).
**Figure S5:** Double positive (DP) cells analyzed by image flow cytometry in young and old human organoids.
**Figure S6:** (A) levels of miR‐29 and miR‐124 with Scramble or miR‐152 mimic transfection. (B) Densitometric analysis of immunoblots shown in Figure 6D. (C) Levels of PHB1 mRNA with Scramble or miR‐152 mimic transfection. (D) Level of miR‐29 with Scramble or miR‐29 mimic transfection (left) and expression of mitochondrial proteins (right). (E) Level of miR‐124 with Scramble or miR‐124 mimic transfection (left) and expression of mitochondrial proteins (right).
**Figure S7:** (A) Divergent PCR to specifically identify *circHIPK3* in human and mouse tissue samples. (B) Levels of circZNF609 in tissues described in Figure 7B. (C) Levels of circZNF609 in cells described in Figure 7C. (D) Seahorse analysis in cells with or without *circHIPK3* silencing.

## Data Availability

The data that supports the findings of this study are available in the Supporting Information of this article.
