# Supplementary material for: Age‐Associated Impairment of Paneth Cells Driven by microRNA‐152 Promotes Intestinal Epithelial Vulnerability to Pathological Stress
Source: Aging Cell. 2026 May 13;25(5):e70542. doi: 10.1111/acel.70542 (PMC13169495; doi:10.1111/acel.70542)
Supplement: Supplementary file 1 — Figure S1: (A) Goblet cell population in small intestines from individuals described in Figure 1. (B) H&E and Mucin2 staining in colons from same individuals described in Figure 1. (C) Goblet cell population in colons from individuals described in Figure 1. Figure S2: (A) Lysozyme positive cells sorted by Imagestream flow cytometry. The organoids were maintained either in growth medium (OGM) or differentiation medium (ODM) as described in Figure 2C. The experiment was repeated 3 times. (B) Percentile of Caspase3 positive cells in the organoids described in Figure 2D. The Values are the means ± SEM (n = 3). Figure S3: (A) Small intestinal (a) H&E staining and measurements (b) permeability assay with FITC‐dextran (c) intercellular junction protein expressions (d) mucin2 positive cell population in young (8‐wks) and aging (76‐wks) mice quantified as mucin2+ cells per 100 cells (DAPI+) in crypt‐villus units. (B) Colonic (a) H&E staining (b) mucin2 staining (c) mucin2 positive cell population in mice described in (A). Figure S4: Time course study of colorimetric absorbance of (A) Complex I and (B) Complex IV activity in small intestinal epithelium of young and aging mice. Values are the means ± SEM (n = 5). Figure S5: Double positive (DP) cells analyzed by image flow cytometry in young and old human organoids. Figure S6: (A) levels of miR‐29 and miR‐124 with Scramble or miR‐152 mimic transfection. (B) Densitometric analysis of immunoblots shown in Figure 6D. (C) Levels of PHB1 mRNA with Scramble or miR‐152 mimic transfection. (D) Level of miR‐29 with Scramble or miR‐29 mimic transfection (left) and expression of mitochondrial proteins (right). (E) Level of miR‐124 with Scramble or miR‐124 mimic transfection (left) and expression of mitochondrial proteins (right). Figure S7: (A) Divergent PCR to specifically identify circHIPK3 in human and mouse tissue samples. (B) Levels of circZNF609 in tissues described in Figure 7B. (C) Levels of circZNF609 in cells described in Figur [file ACEL-25-e70542-s001.pdf]

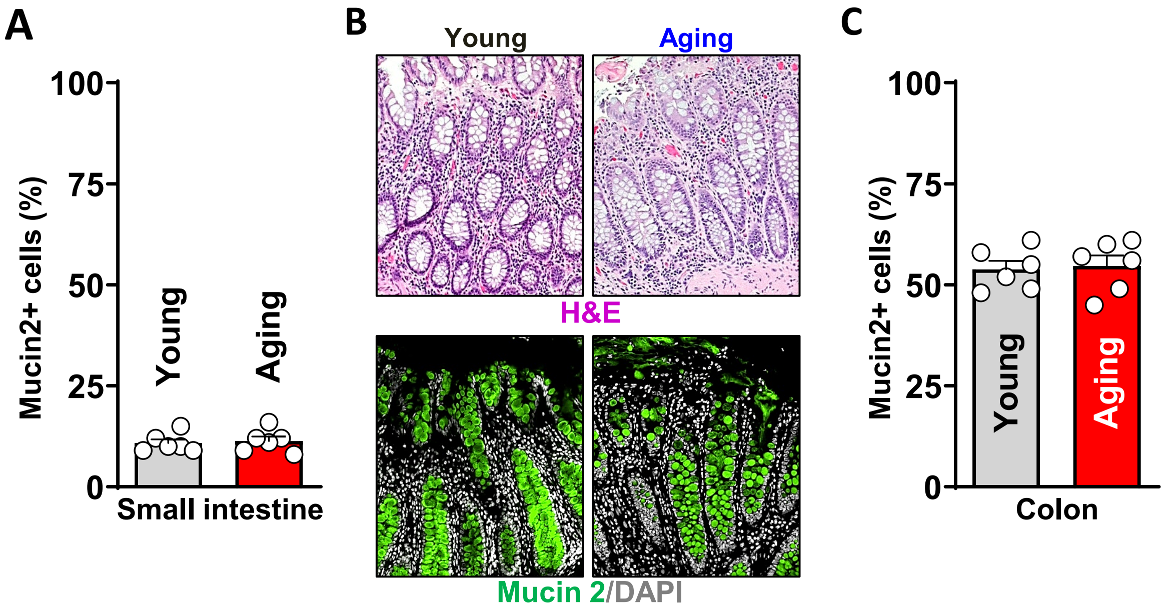

Figure S1: **(A)** Goblet cell population in small intestines from individuals described in Fig 1. (**B**) H&E and Mucin2 staining in colons from same individuals described in Fig 1. **(C)** Goblet cell population in colons from individuals described in Fig 1.

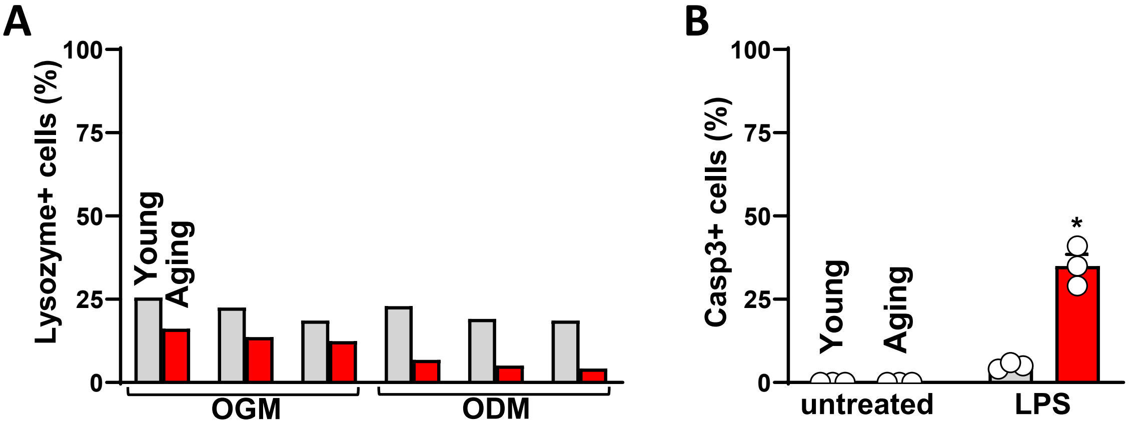

Figure S2: **(A)** Lysozyme positive cells sorted by Imagestream flow cytometry. The organoids were maintained either in growth medium (OGM) or differentiation medium (ODM) as described in Fig 2C. The experiment was repeated 3 times. **(B)** Percentile of Caspase3 positive cells in the organoids described in Fig 2D. The Values are the means ± SEM (n = 3).

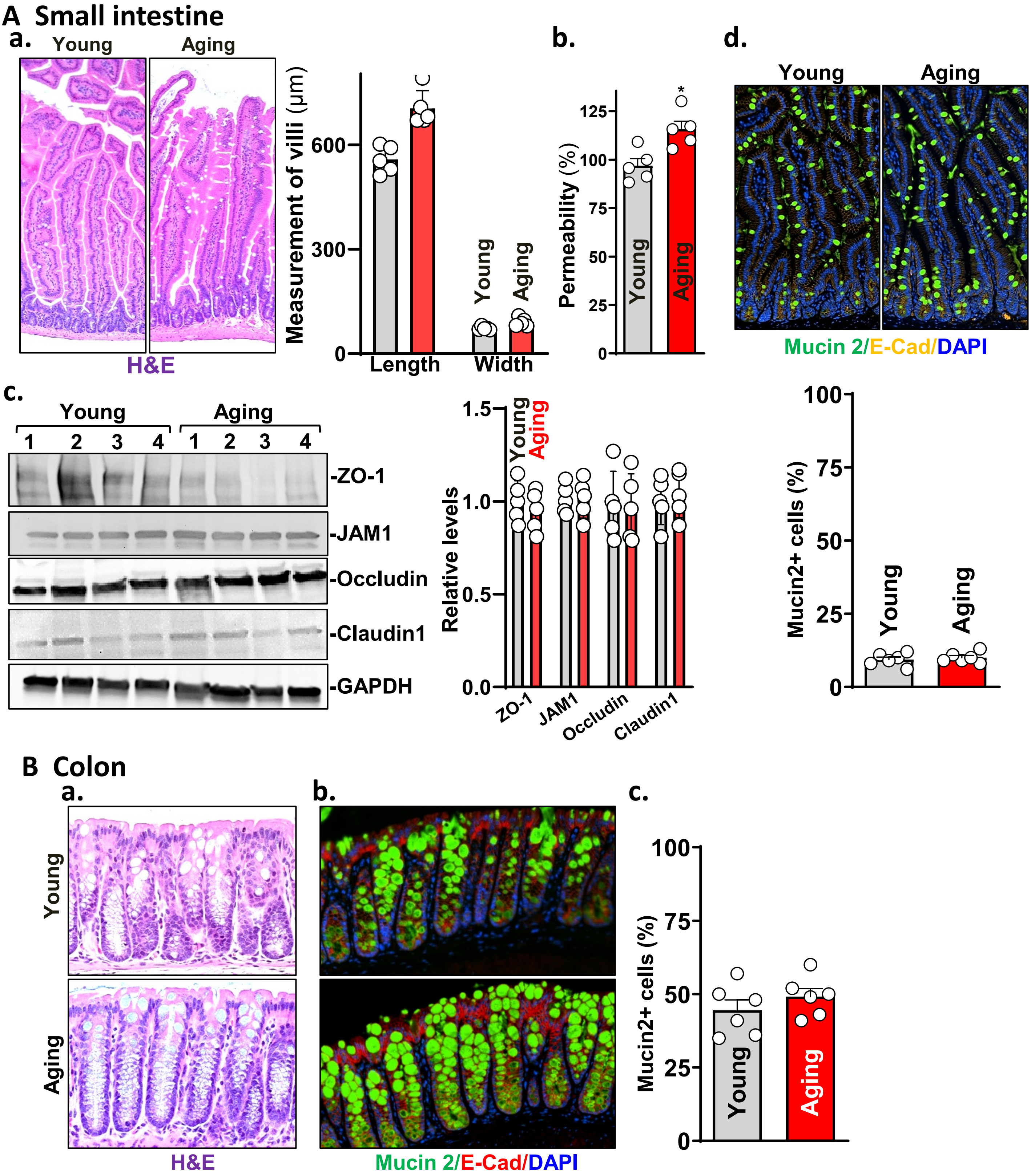

Figure S3: **(A)** Small intestinal (a) H&E staining and measurements (b) permeability assay with FITC-dextran (c) intercellular junction protein expressions with densitometric analysis (d) mucin2 positive cell population in young (8-wks) and aging (76-wks) mice quantified as mucin2+ cells per 100 cells (DAPI+) in crypt-villus units. (**B**) Colonic (a) H&E staining (b) mucin2 staining (c) mucin2 positive cell population in mice described in (A).

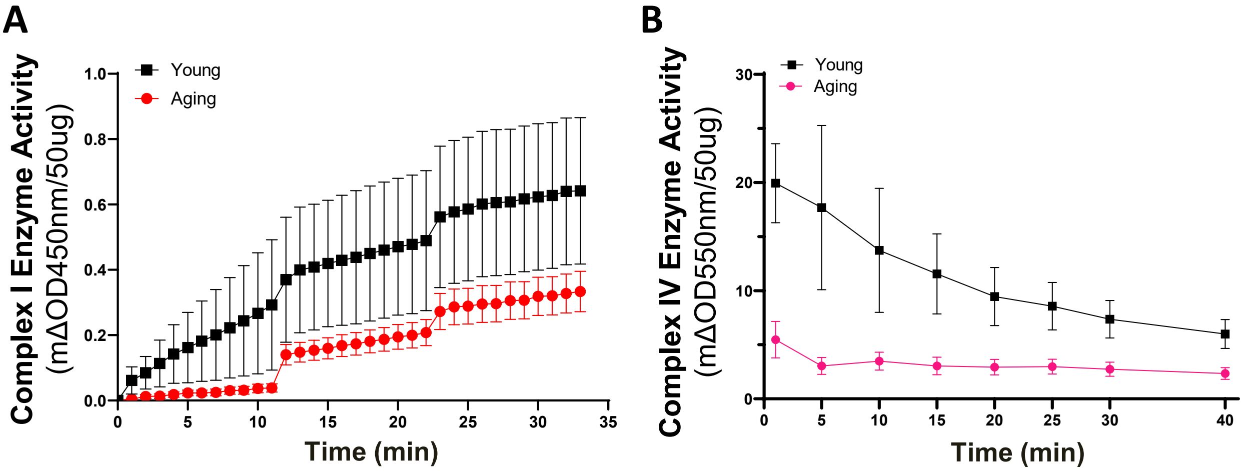

Figure S4: Time course study of colorimetric absorbance of **(A)** Complex I and **(B)** Complex IV activity in small intestinal epithelium of young and aging mice. Values are the means ± SEM (n = 5).

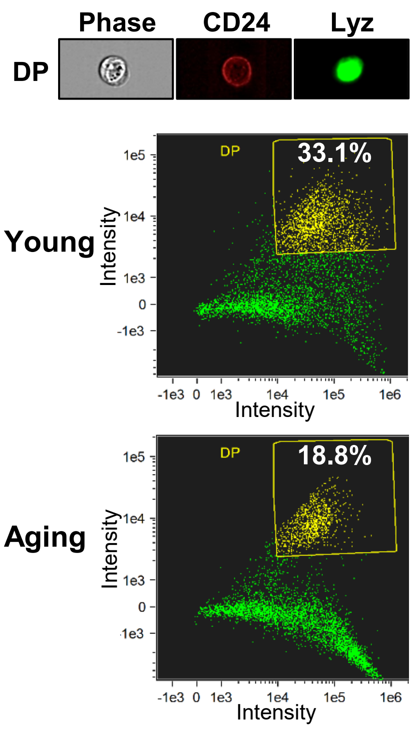

Figure S5: Double positive (DP) cells analyzed by image flow cytometry in young and old human organoids.

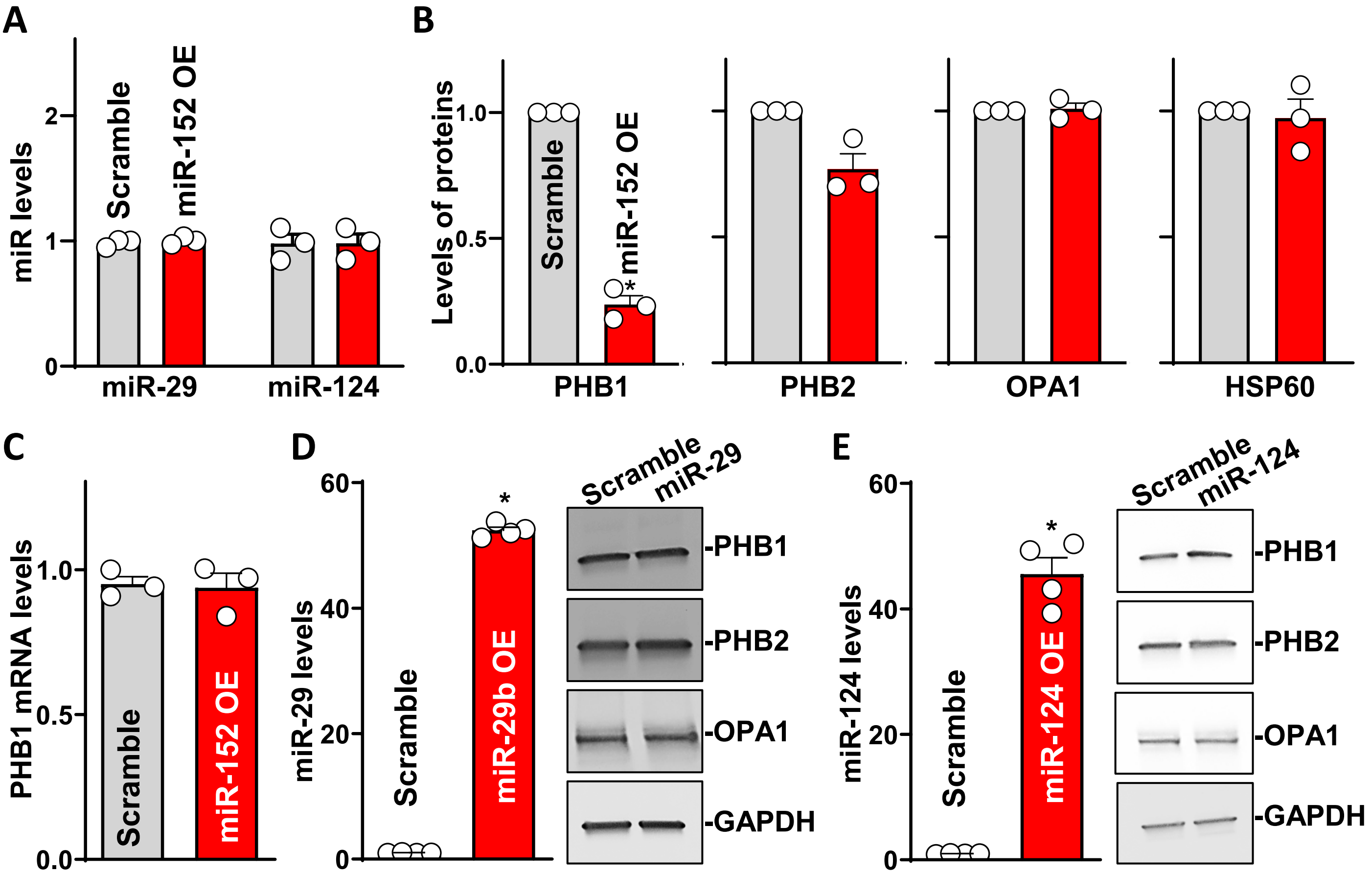

Figure S6: **(A)** levels of miR-29 and miR-124 with Scramble or miR-152 mimic transfection. **(B)** Densitometric analysis of immunoblots shown in Fig 6D. **(C)** Levels of PHB1 mRNA with Scramble or miR-152 mimic transfection. **(D)** Level of miR-29 with Scramble or miR-29 mimic transfection (left) and expression of mitochondrial proteins (right). **(E)** Level of miR-124 with Scramble or miR-124 mimic transfection (left) and expression of mitochondrial proteins (right).

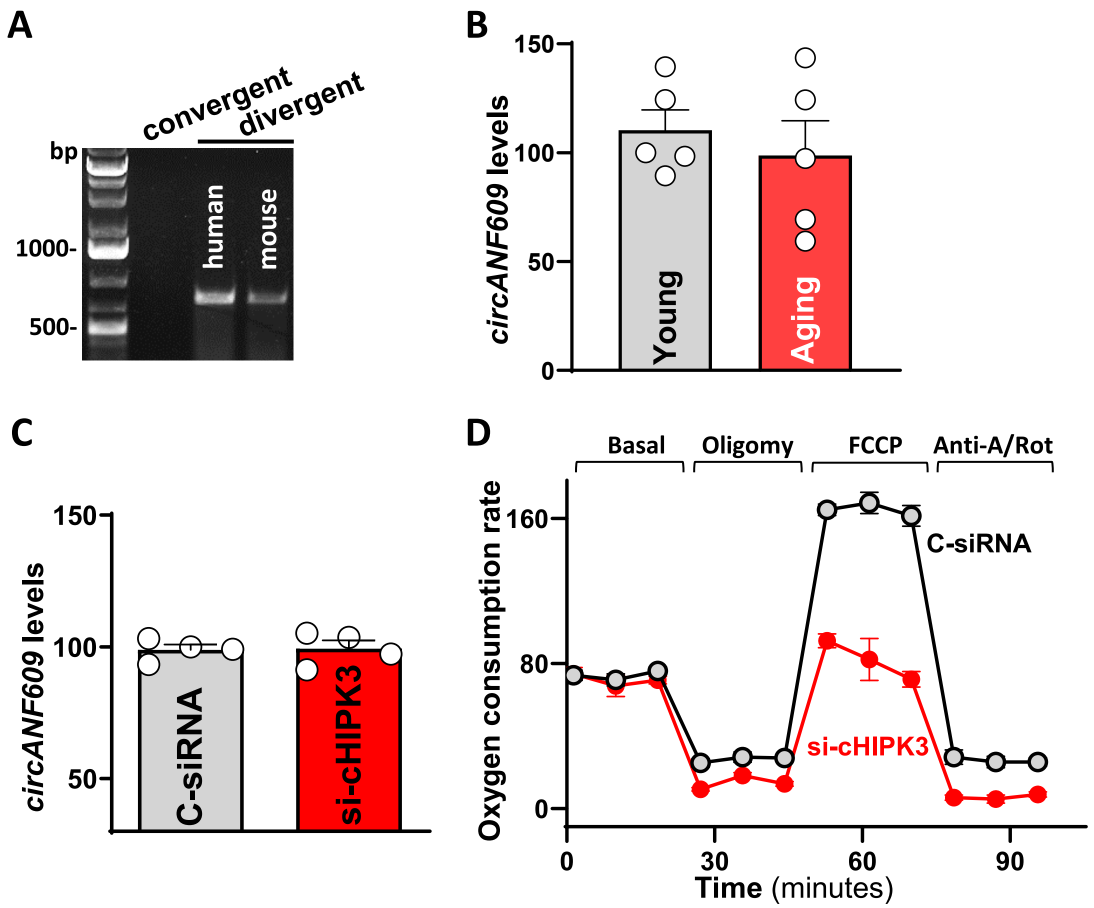

Figure S7: **(A)** Divergent PCR to specifically identify *circHIPK3* in human and mouse tissue samples. **(B)** Levels of circZNF609 in tissues described in Fig. 7B. **(C)** Levels of circZNF609 in cells described in Fig. 7C. **(D)** Seahorse analysis in cells with or without *circHIPK3* silencing.
